# Quaternary Structure, Salt Sensitivity, and Allosteric Regulation of β-AMYLASE2 From *Arabidopsis thaliana*

**DOI:** 10.3389/fpls.2018.01176

**Published:** 2018-08-14

**Authors:** Jonathan D. Monroe, Lauren E. Pope, Jillian S. Breault, Christopher E. Berndsen, Amanda R. Storm

**Affiliations:** ^1^Department of Biology, James Madison University, Harrisonburg, VA, United States; ^2^Department of Chemistry and Biochemistry, James Madison University, Harrisonburg, VA, United States; ^3^Department of Biology, Western Carolina University, Cullowhee, NC, United States

**Keywords:** β-amylase, *Arabidopsis thaliana*, allosteric regulation, starch, sigmoidal kinetics, secondary binding site, KCl

## Abstract

The β-amylase family in *Arabidopsis thaliana* has nine members, four of which are both plastid-localized and, based on active-site sequence conservation, potentially capable of hydrolyzing starch to maltose. We recently reported that one of these enzymes, BAM2, is catalytically active in the presence of physiological levels of KCl, exhibits sigmoidal kinetics with a Hill coefficient of over 3, is tetrameric, has a putative secondary binding site (SBS) for starch, and is highly co-expressed with other starch metabolizing enzymes. Here we generated a tetrameric homology model of Arabidopsis BAM2 that is a dimer of dimers in which the putative SBSs of two subunits form a deep groove between the subunits. To validate this model and identify key residues, we generated a series of mutations and characterized the purified proteins. (1) Three point mutations in the putative subunit interfaces disrupted tetramerization; two that interfered with the formation of the starch-binding groove were largely inactive, whereas a third mutation prevented pairs of dimers from forming and was active. (2) The model revealed that a 30-residue N-terminal acidic region, not found in other BAMs, appears to form part of the putative starch-binding groove. A mutant lacking this acidic region was active and did not require KCl for activity. (3) A conserved tryptophan residue in the SBS is necessary for activation and may form π-bonds with sugars in starch. (4) Sequence alignments revealed a conserved serine residue next to one of the catalytic glutamic acid residues, that is a conserved glycine in all other active BAMs. The serine side chain points away from the active site and toward the putative starch-binding groove. Mutating the serine in BAM2 to a glycine resulted in an enzyme with a *V*_Max_ similar to that of the wild type enzyme but with a 7.5-fold lower *K*_M_ for soluble starch. Interestingly, the mutant no longer exhibited sigmoidal kinetics, suggesting that allosteric communication between the putative SBS and the active site was disrupted. These results confirm the unusual structure and function of this widespread enzyme, and suggest that our understanding of starch degradation in plants is incomplete.

## Introduction

Starch is the principle storage form of reduced carbon and energy in plants and it accumulates in plastids for short-term or long-term requirements. Starch granules are composed primarily of amylopectin, which contains linear chains of α-1,4-linked glucose with regularly spaced α-1,6 branches (Zeeman et al., 2010). Amylose chains composed of mostly α-1,4-linked glucose make up a smaller portion of the granule. The branching architecture of starch granules has been optimized by evolution to allow starch granules to grow to a massive size (Ball et al., 2011). In chloroplasts β-amylase (BAM) enzymes play an important role in starch degradation hydrolyzing the penultimate α-1,4-glycoside linkage from the non-reducing ends of starch (Scheidig et al., 2002; Fulton et al., 2008).

Of the nine BAM genes in *Arabidopsis thaliana*, four encode plastid localized, catalytically active enzymes (BAM1, BAM2, BAM3, and BAM6) (for citations see Monroe et al., 2017). BAM5 is catalytically active, cytosolic and expressed in phloem tissue. Two catalytically inactive BAMs are plastid localized (BAM4 and BAM9) and probably play a regulatory role in starch degradation, and two are nuclear-localized transcription factors (BAM7 and BAM8).

Analysis of the primary structure and conservation of intron positions of BAM proteins in land plants revealed two groups of *BAM* genes that we designated Subfamily I and Subfamily II, that diverged prior to the origin of land plants (Monroe et al., 2017). The ancestral members of these subfamilies, still present in bryophytes today, resemble BAM2 and BAM3. BAM3, a member of Subfamily I, has received the most attention because it plays a prominent role in leaf starch degradation (Lao et al., 1999; Kaplan and Guy, 2005; Fulton et al., 2008; Monroe et al., 2014). In contrast, despite its ancient origin, the physiological function of BAM2, the founding member of Subfamily II, is presently unknown. T-DNA knockout Arabidopsis mutants lacking BAM2 did not accumulate any measurable leaf starch at 4 weeks of age (Fulton et al., 2008), however, in 8-week old plants there was an accumulation of iodine-stained starch (Monroe et al., 2014). Also, the *BAM2* gene is co-expressed with most of the other proteins involved in starch degradation suggesting that it is likely to play a role in the process (Monroe et al., 2017).

The first high-resolution structural characterization of a plant BAM was from soybean (*Glycine max*) (Mikami et al., 1993). The soybean BAM is an ortholog of Arabidopsis BAM5, which, like BAM2, is a member of Subfamily II. This and subsequent reports established that the enzyme is a monomeric, ca. 50 kDa (β/α)_8_ TIM-barrel protein with a deep catalytic cleft where two glutamic acid residues act as a general acid and base during hydrolysis (Mikami et al., 1994). A sweet potato (*Ipomea batatas*) BAM was crystallized as a tetramer, but the active form was monomeric (Thoma et al., 1963; Cheong et al., 1995). Laederach et al. (1999) identified all of the active-site residues in the soybean protein that form H-bonds with starch. When aligned with this soybean BAM, the Arabidopsis BAMs that were reported to be catalytically active shared all of these conserved, active site residues, whereas the Arabidopsis BAMs that were reported to be catalytically inactive contained some changes among these residues (Monroe et al., 2017).

BAM2 was initially reported to be catalytically inactive (Fulton et al., 2008; Li et al., 2009). However, BAM2’s conservation of active site residues led us to investigate conditions that might reveal BAM2 activity and we learned that, unlike all other characterized BAMs, BAM2 was inactive without added salts (Monroe et al., 2017). This requirement is likely met *in vivo* by physiological concentrations of K^+^, which are typically greater than 120 mM in the stroma (Finazzi et al., 2015). BAM2 activity was saturated by about 80 mM KCl (Monroe et al., 2017). Monovalent cations can affect enzymes in two ways: they can interact with the substrate directly (Type I interaction), or they can indirectly change the structure of the active site of the enzyme (Type II interaction) (Di Cera, 2006). Because BAM2 was activated by a variety of monovalent cations the interaction is believed to be Type II (Monroe et al., 2017).

The discovery that BAM2 required K^+^ for activity allowed characterization of enzyme kinetics and it was determined that BAM2 had sigmoidal kinetics with a Hill coefficient of over 3 (Monroe et al., 2017), suggesting that the protein might be multimeric and/or rely on an allosteric effector. Size-Exclusion Chromatography Multi-Angle Light Scattering (SEC-MALS) was then used to determine that BAM2 was a tetramer. Analysis of alignments of BAM2 proteins from land plants revealed a large, conserved, solvent-exposed surface centered on α-helix 6 (numbering from Mikami et al., 1993) that we proposed might be a secondary binding site (SBS) for starch, and that starch-binding to the SBS might somehow influence the active site. Support for this hypothesis was obtained by replacing two conserved glycine residues with bulky methionine residues in the SBS resulting in proteins with up to 95% less activity than the wild type (Monroe et al., 2017).

The ability to study BAM2 activity allowed the discovery of its many unusual catalytic properties; we wanted to explore the structural basis for some these properties. Here we describe a tetrameric homology model of Arabidopsis BAM2 that we used to identify potentially key structural and regulatory residues. We then used site directed mutagenesis to reveal that activity requires that the protein be at least a dimer with an intact SBS. We identified a uniquely conserved acidic region that is involved in the salt requirement, and several residues that play essential roles in allosteric regulation of BAM2.

## Materials and Methods

### Modeling

The structure of BAM2 was modeled using I-TASSER (Yang et al., 2015) and equilibrated in YASARA (Krieger and Vriend, 2014). The BAM2 model was then aligned with the coordinates of sweet potato β-amylase tetramer (PDB ID 1FA2) in YASARA (Cheong et al., 1995). The average RMSD of the backbone atoms between the BAM2 model and the individual chains of the 1FA2 tetramer was 0.8 Å. The BAM2 tetramer model was then energy minimized using an AMBER14 force field to improve bond lengths and amino acid geometry while reducing clashes between atoms.

### Mutagenesis, Protein Expression and Purification

Construction of the WT BAM2 expression vector in pETDuet-1 was previously described (Monroe et al., 2017). Amino acid changes were made in the WT sequence using the QuikChange mutagenesis strategy (Agilent Technologies). Mutagenesis primers contained the desired mutation with about ∼20 nucleotides on either side that were complementary to the WT sequence. Primer sequences for making amino acid changes were as follows: B2_W456A, 5′-GCAGGTGCTGAATGCTGCTGCGGATGCTAGTATACCTGTTGC-3′, B2_D490R, 5′-CAAAGCCCCTTACCGATCCTCGTGGTCGCCACCTTTCATGTTTC-3′, B2_F238A, 5′-GACTGCTCTTGAGGTTTACGCTGATTACATGAGAAGCTTTCG-3′, B2_W449A, 5′- CAGATCCAGAAGGTCTAGTTGCGCAGGTGCTGAATGCTGCTTG-3′, and B2_S464G, 5′- GATGCTAGTATACCTGTTGCTGGTGAAAATGCTCTTCCTTGTTATG-3′, along with their complementary sequences. To amplify the entire plasmid PCR conditions were as follows: 95°C for 5 min; cycle of 95°C for 30 s, 60°C for 30 s, and 72°C for 4 min repeated 20 times; and a final extension at 72°C for 10 min. The PCR products were digested with DpnI for 1.5 h at 37°C and then transformed into competent DH5α *Escherichia coli* cells. To create an N-terminal deletion mutant (NDel), a BamHI site was introduced into the WT sequence in frame between the acidic region and the catalytic domain. A BamHI site already existed in the vector between the encoded N-terminal His-tag and the start of the BAM2 coding region. The primers used for PCR were B2_NDel, 5′-TGATGAAGAAATTGTGCAGGATCCAGAGCGTGATTTTGCTGGC-3′ and the complementary sequence. The protocol for the PCR was the same as for mutagenesis described above. The resulting DNA was then digested with both DpnI for 1.5 h at 37°C, and then BamHI for 4 h at 37°C. After digestion, the DNA was ligated and then transformed into competent DH5α *E. coli* cells. All of the nucleotide changes were confirmed by sequencing at Eurofins Genomics. Each plasmid was then transformed into BL21+ *E. coli* cells, and proteins were expressed and purified as described by Monroe et al. (2014). The concentration of the purified proteins was determined using the Bio-Rad Protein Assay Kit with BSA as the standard.

### Size-Exclusion Chromatography Multi-Angle Light Scattering (SEC-MALS)

Size-Exclusion Chromatography Multi-Angle Light Scattering was performed as described previously (Monroe et al., 2017). Proteins at a concentration of 1–2 mg mL^−1^ were injected into a 4.6- × 300-mm size-exclusion column with a particle size of 5 μm and a pore size of 300 Å at a flow rate of 0.5 mL min^−1^ in filter-sterilized (0.2 mm) 10 mM MOPS, pH 7, 250 mM KCl. Absorbance data were collected on an Agilent G1315B Diode Array detector at 280 and 212 nm, and light scattering was measured on a miniDAWN-TREOS (Wyatt Technologies). Data were analyzed using ASTRA (version 6.1.5.22). The predicted molecular mass and extinction coefficient of each monomer were determined using the Protein Identification and Analysis Tools on the ExPASy Server (Gasteiger et al., 2005).

### Enzyme Assays

Purified enzymes were diluted with 50 mM MOPS, pH 7, containing 1 mg mL^−1^ porcine gelatin. Amylase assays were conducted in 0.5 mL containing 50 mM MES (pH 6) and various amounts of soluble starch (Acros Organics). Unless noted, assays also included 100 mM KCl. Reactions were stopped after 20 min by immersion in a boiling water bath for 3 min; then, reducing sugars were measured by the Somogyi-Nelson assay (Nelson, 1944) with maltose as the standard. *V*_Max_, *K*_M_, and Hill coefficients were determined by fitting the data to the Hill equation using Excel Solver (Gadagkar and Call, 2015).

## Results

Compared with other well-known β-amylases, BAM2 in Arabidopsis has some very unusual structural and catalytic properties. While all other active BAMs are monomeric, display hyperbolic kinetics, and do not have a known salt requirement, BAM2 is a tetramer, possesses a putative SBS for starch, requires K^+^ for activity, and has sigmoidal kinetics. However, its physiological function is not known. Understanding more about its structure may lead to new hypotheses regarding its role in plants so we generated a model structure for the BAM2 tetramer and tested the importance of various structural features for conferring some of the unique functional characteristics.

### Constructing a Tetramer Model of BAM2

In the absence of a crystal structure of Arabidopsis BAM2, we constructed a model of a BAM2 tetramer. Starting with the primary sequence of Arabidopsis BAM2 (NP_191958.3) lacking its predicted 55 amino acid N-terminal chloroplast transit peptide (Fulton et al., 2008) we used I-TASSER (Yang et al., 2015) to generate a homology model of the monomer based on the structure of BAM5 from soybean. The monomer model was then used to build a tetramer model based on the homo-tetrameric crystal structure of sweet potato BAM5 (PDB ID 1FA2) (Cheong et al., 1995).

**FIGURE 1:**
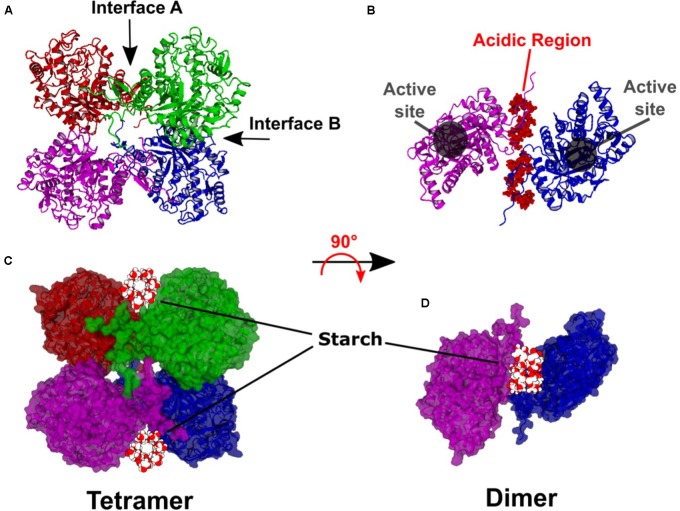
Hypothetical model of the Arabidopsis BAM2 tetramer. **(A)** Ribbon structure of the BAM2 tetramer indicating Interface A and B. **(B)** Ribbon structure of a BAM2 dimer (rotated 90° from **A**) illustrating the active sites and the location of the N-terminal acidic region of each subunit. **(C)** Same orientation as **A** but in space-filling view with a 13-residue segment of V-amylose (based on PDB ID 1C58) in the starch-binding grooves of each dimer. **(D)** Space-filling view of **B** with the segment of V-amylose in the starch-binding groove.

The resulting model has dihedral symmetry and forms an apparent “dimer of dimers” with the primary dimer consisting of two BAM subunits interacting back-to-back (Interface A) to bring together the SBSs. Then two of these dimers pair together to form the tetramer (Interface B) in a bottom-to-bottom manner (**Figure 1A**). Interface A involves a relatively long loop between α-helix 7 and β-sheet 8 identified as L’7 (Mikami et al., 1993). Interface B involves the N-terminal ends of α-helices 3 and 4. The active sites of each subunit open to the solvent and are ∼20 Å from Interface A and ∼18 Å from Interface B (**Figure 1B**). Moreover, the active sites do not contain amino acids associated with either Interface. The N-terminal and C-terminal regions of each subunit are not integral to the TIM barrel but associate with each other and extend ∼90° from the primary dimer axis (**Figure 1B**).

Each primary dimer creates a deep groove between the subunits, lined on each side by conserved surface residues previously identified as the SBS (Monroe et al., 2017). The groove has a surface area of ∼9000 Å^2^ and a volume of ∼6000 Å^3^. The groove width ranges between 18 and 37 Å and is about 35 Å in length at its longest point. The groove is longer if the N and C termini of each subunit are included. A starch chain such as the 13-residue segment of V-amylose modeled in **Figures 1C,D** (PDB ID 1C58) with a diameter of 12 Å might be expected to sit on top of these two extensions within what we now refer to as the “starch-binding groove.”

### BAM2 Activity Requires a Dimer With an Intact Interface A

**FIGURE 2:**
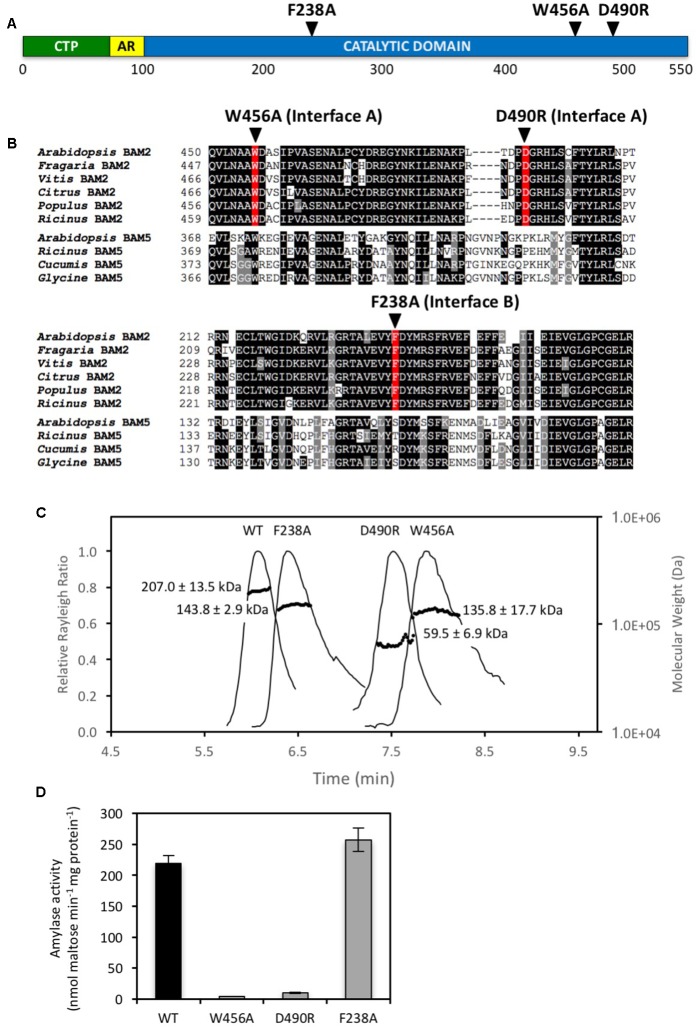
Locations and properties of three mutations in the subunit interfaces of the BAM2 tetramer. **(A)** Map of the primary structure of BAM2 with the relative positions of three point mutations in the subunit interfaces, as predicted from the tetramer model. CTP indicates chloroplast transit peptide. AR indicates acidic region. **(B)** Portion of an alignment of six BAM2 ortholog sequences (*Arabidopsis thaliana*, NP_191958.3; *Fragaria vesca* subsp. *vesca*, XP_004306786.1; *Vitis vinifera*, XP_002274612.2; *Citrus clementina*, XP_006445046.1; *Populus trichocarpa*, XP_002320794.2; and *Ricinus communis*, XP_002511858.1) with four BAM5 sequences (*Arabidopsis thaliana*, NP_567460.1; *Ricinus communis*, XP_002515712.1; *Cucumis sativus*, XP_011658713.1; and *Glycine max*, NP_001236247.1) showing the locations of the three point mutations. **(C)** SEC-MALS elution profiles (representative) and masses of wild-type BAM2 and three BAM2 mutants. Purified proteins were in 10 mM MOPS, pH 7, and 250 mM KCl. Masses are averages of each protein ± SD (*n* = 3). **(D)** Catalytic activity of wild-type (WT) BAM2 and three mutants of BAM2 with point mutations in putative interface residues. Assays were conducted with 80 mg mL^−1^ soluble starch and 100 mM KCl. Bars represent activity ± SD (*n* = 3).

In order to test the validity of the tetrameric homology model of BAM2, several conserved residues located in the interfaces were mutated. To disrupt Interface A, Trp456 was changed to Ala to eliminate its involvement in hydrophobic interactions, and Asp490 was changed to Arg to reverse the charge (**Figure 2A**). Trp456 is also conserved in BAM5 but it is in a poorly conserved region, and Asp490 is not conserved in BAM5 (**Figure 2B**). After sequencing each plasmid to confirm the changes, proteins were expressed in *E. coli* and purified using their N-terminal 6-His tags to near homogeneity as previously described (Monroe et al., 2017). We used SEC-MALS to measure the mass of each native protein. The predicted mass of the native WT BAM2 monomer without its N-terminal chloroplast transit peptide is 57.1 kDa whereas the calculated mass of the purified WT protein using SEC-MALS was 207.0 ± 13.5 kDa (**Figure 2C**), which is ∼3.6 times larger than the predicted mass and consistent with BAM2 forming a tetramer. The W456A mutant had a calculated mass of 135.8 ± 17.7 kDa, and the D490R mutant had a calculated mass of 59.5 ± 6.9 kDa indicating that they were a dimer (2.4:1) and monomer (1.04:1), respectively (**Figure 2C**). When assayed with 80 mg/mL soluble starch, both of the mutants with changes in Interface A had less than 5% of the WT activity (**Figure 2D**).

To disrupt Interface B Phe238 was changed to Ala, which, like the mutated Interface A residues, is also in a highly conserved region of BAM2 (**Figure 2B**). SEC-MALS analysis indicated that the F238A mutant had a mass of 143.8 ± 2.9 kDa which is consistent with it being a dimer (2.51:1) (**Figure 2C**). Unlike the mutations that disrupted Interface A, however, the F238A mutant had activity that was similar to that of the WT protein (**Figure 2D**).

### An N-terminal Acidic Region Is Necessary for BAM2’s K^+^ Requirement

**FIGURE 3:**
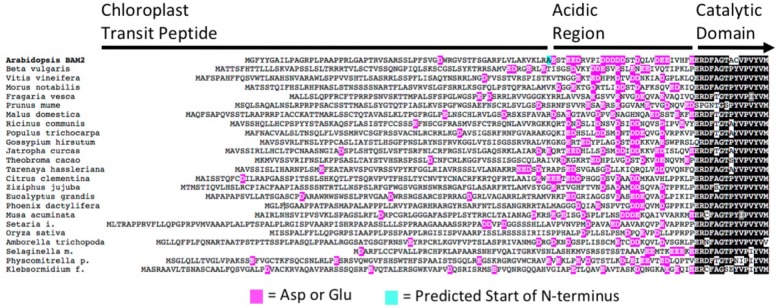
Alignment of the N-terminal portions of 24 BAM2 orthologs from land plants and the charophyte alga *Klebsormidium nitens.* Glutamic acid and aspartic acid residues are highlighted in magenta, and the predicted N-terminal residue of the mature Arabidopsis protein is highlighted in blue. Sequences used were *Amborella trichopoda*, XP_006827627.2; *Arabidopsis thaliana*, NP_191958.3; *Beta vulgaris* subsp. *Vulgaris*, XP_010670423.1; *Citrus clementina*, XP_006445046.1; *Eucalyptus grandis*, XP_010055131.1; *Fragaria vesca* subsp. *Vesca*, XP_004306786.1; *Gossypium hirsutum*, XP_016694835.1; *Jatropha curcas*, XP_012083395.1; *Klebsormidium nitens*, kfl00081_0270; *Malus domestica*, XP_008338858.1; *Morus notabilis*, XP_010105936.1; *Musa acuminata* subsp. *Malaccensis*, XP_009392820.1; *Oryza sativa* Japonica Group, XP_015611670.1; *Phoenix dactylifera*, XP_008787503.1; *Physcomitrella patens*, XP_024360526.1; *Populus trichocarpa*, XP_002320794.2; *Prunus mume*, XP_008232901.1; *Ricinus communis*, XP_015584439.1; *Selaginella moellendorffii*, XP_024540821.1; *Setaria italica*, XP_012699441.2; *Tarenaya hassleriana*, XP_010540283.1; *Theobroma cacao*, XP_017981267.1; *Vitis vinifera*, XP_002274612.2; and *Ziziphus jujuba*, XP_015868824.1.

Unlike all other characterized BAM proteins, BAM2 requires K^+^ for activity, a requirement likely met *in vivo* by physiological concentrations of K^+^ (Finazzi et al., 2015). In looking for a potential site for cation interaction, a distinctive N-terminal region was observed in BAM2. Between the predicted N-terminus of mature BAM2 and the conserved catalytic domain is a region of 30 amino acids, 14 of which are either Glu or Asp and would be expected to be negatively charged at physiological pH (**Figure 3** and modeled in **Figure 1B**). A sequence alignment of 24 BAM2 orthologs from flowering plants, a seedless vascular plant, a non-vascular plant and a charophyte alga revealed that all of these sequences had a similar region that contained a high frequency of acidic residues, although they did not align well in the traditional sense (**Figure 3**). We refer to this region as the “acidic region.”

**FIGURE 4:**
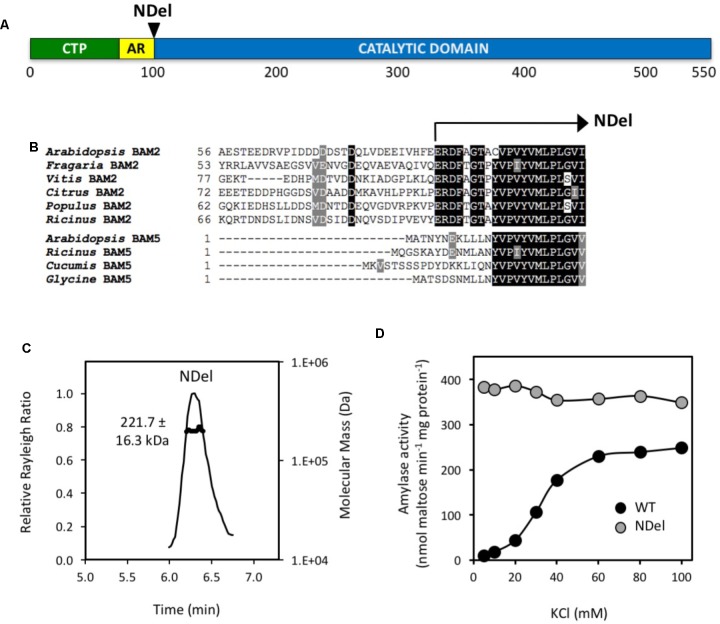
Location and properties of an N-terminal deletion mutant (NDel) of BAM2. **(A)** Map of the primary structure of BAM2 with the relative position of the N-terminal deletion, NDel. **(B)** Portion of an alignment of six BAM2 sequences with four BAM5 sequences (identified in **Figure 2**) showing the location of the N-terminal deletion. **(C)** SEC-MALS elution profile (representative) and mass of the NDel mutant protein. Purified protein was in 10 mM MOPS, pH 7, and 250 mM KCl. Mass is the average ± SD (*n* = 3). **(D)** Catalytic activity of wild-type (WT) BAM2 (black circles) and the NDel mutant (gray circles) assayed with 80 mg mL^−1^ soluble starch and variable concentrations of KCl.

To test the importance of the acidic region and its potential role in the salt requirement of BAM2 activity, we generated a mutant lacking the first 30 amino acids following the predicted chloroplast transit peptide (**Figures 4A,B**). Despite the fact that this region appears to be located near both interfaces in our model (**Figure 1**), SEC-MALS analysis indicated that the mutant protein had a mass of 221.7 ± 16.3 kDa and thus was tetrameric (3.9:1) (**Figure 4C**). Activity assays showed that the NDel mutant had higher catalytic activity than the WT. Interestingly, the activity of the mutant was largely unaffected by the concentration of KCl (**Figure 4D**).

### Allosteric Activation of BAM2 Involves Trp449 and Ser464

**FIGURE 5:**
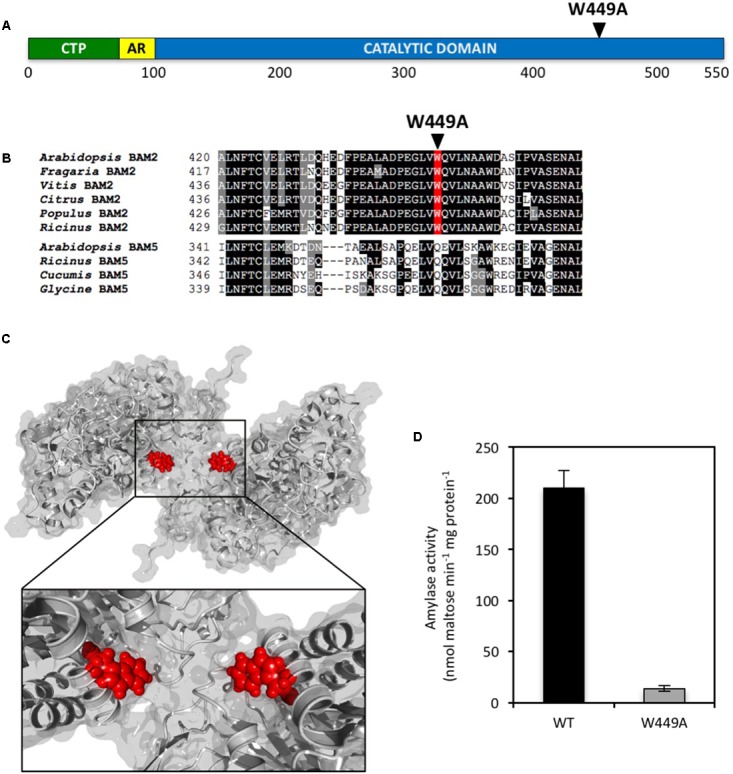
Location and properties of a point mutation in the putative SBS of BAM2. **(A)** Map of the primary structure of BAM2 with the relative position of the W449A point mutation in the SBS. **(B)** Portion of an alignment of six BAM2 sequences with four BAM5 sequences (identified in **Figure 2**) showing the location of the W449A mutation. **(C)** Model of a BAM2 dimer illustrating the location of W449 in the starch-binding groove. **(D)** Catalytic activity of wild-type BAM2 and the W449A mutant. Assays were conducted with 80 mg mL^−1^ soluble starch and 100 mM KCl. Bars represent activity ± SD (*n* = 3).

The presence of a large, conserved region on the surface of BAM2 ∼20 Å from the active site led us to suspect that it might be a SBS that is involved in allosteric regulation of the protein. Two Gly-to-Met mutations in this region were previously made to attempt to interfere with binding at this site. Both of the mutant proteins were found to be tetramers with very little activity compared with the WT protein in agreement with this hypothesis (Monroe et al., 2017). Examination of the tetramer model revealed a conserved Trp449 residue near the center of the putative SBS (**Figures 5A–C**) that we hypothesize might be involved in aromatic-sugar stacking (π-interactions) (Asensio et al., 2013). To test this hypothesis, we mutated Trp449 to Ala and the resulting protein had only 7% of the WT activity at 80 mg/mL soluble starch (**Figure 5D**). The functional requirement of key residues in the SBS support its role as an allosteric binding site.

**FIGURE 6:**
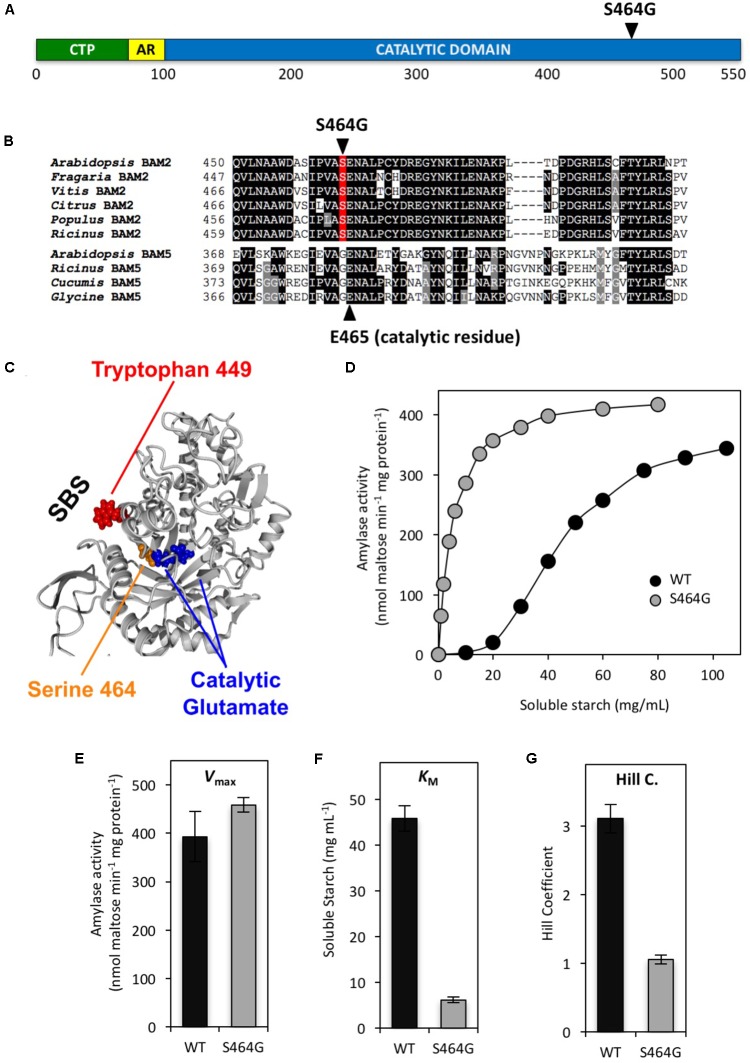
Location and properties of a mutation in the putative allosteric network of BAM2. **(A)** Map of the primary structure of BAM2 with the relative position of the S464G mutation. **(B)** Portion of an alignment of six BAM2 sequences with four BAM5 sequences (identified in **Figure 2**) showing the location of S464G and the catalytic Glu465. **(C)** Model of a BAM2 monomer illustrating the location of S464 adjacent to the catalytic Glu465 and the position of the SBS. **(D)** Catalytic activity of wild-type (WT) BAM2 (black circles) and the S464A mutant (gray circles) assayed with 250 mM KCl and variable concentrations of soluble starch. **(E–G)** Catalytic constants for WT BAM2 and the S464A mutant. Values are means ± SD (*n* = 3). Data from the WT BAM2 in D-G are from Monroe et al. (2017).

We were also interested in how allosteric binding was communicated to the active site. It is unlikely that the mechanism of allosteric activation of BAM2 by the binding of starch to the SBS involves a radical change in the active site as all of the active-site residues identified to interact with the substrate in the soybean BAM5 crystal structure (Laederach et al., 1999) are identical in all the active Arabidopsis BAMs including BAM2 (Monroe et al., 2017). We therefore examined the region located between the active site and the allosteric site for residues that were conserved but unique to BAM2 orthologs and identified Ser464 (**Figures 6A–C**). This residue is a Ser in all of the BAM2 orthologs that were examined, whereas the corresponding residue in all of the other active BAMs is a Gly residue. To determine if this Ser was involved in allosteric regulation of BAM2 we mutated it to a Gly and measured the mutant’s kinetic properties (**Figure 6D**). Remarkably, this mutation had no effect on *V*_Max_ (**Figure 6E**) but the mutant had a *K*_M_ that was 7.5-fold lower than the WT protein (**Figure 6F**), and with a Hill coefficient of 1.06 ± 0.07 the allosteric regulation of the protein was completely disrupted (**Figure 6G**). Thus the S464G mutation suggests a direct structural connection between the SBS and the active site further supporting our tetrameric model of BAM2.

## Discussion

Our approach to understanding the role of BAM2 has been focused on its unusual structural and catalytic properties, having observed that it requires K^+^ for activity, is a tetramer with sigmoidal kinetics, and possesses a putative SBS, presumably for starch (Monroe et al., 2017). These properties make BAM2 highly unusual for a β-amylase and suggest that it might therefore play an unusual role.

Lacking a crystal structure of BAM2, we constructed a tetrameric homology model to attempt to probe the structural basis of some of BAM2’s unusual properties. Crystal structures of several plant BAMs have been solved and all are orthologs of Arabidopsis BAM5 (Mikami et al., 1993; Cheong et al., 1995; Rejzek et al., 2011). Analysis of conserved intron positions and sequence alignments indicated that BAM5 was probably derived from a BAM2-like gene, perhaps near the time of the origin of seed plants making BAM5 and BAM2 much more closely related to each other than either are to BAM3 (Monroe et al., 2017). The crystal structures are useful for generating homology models of the remaining BAMs, including BAM2 (Monroe et al., 2017), as they all share considerable sequence identity. One of the crystal structures, from sweet potato, is a crystallographic tetramer although this enzyme is active as a monomer and shares none of the unusual properties of BAM2 (Cheong et al., 1995).

Using the sweet potato tetramer structure as a template, we positioned four BAM2 monomeric homology models in roughly the same orientation as those in the sweet potato structure, as a dimer of dimers, and noticed that the subunit interfaces contained residues that are highly conserved among BAM2 orthologs. Moreover, the subunit interface in the dimer formed a deep groove with each side of the groove comprised of the large, putative SBS. The groove is large enough to accommodate a starch chain (**Figure 1**). The active sites of each monomer are positioned facing out so that it is unlikely that tetramerization would block access of the substrate to these sites. In addition to the TIM barrels that make up most of the monomeric structure, the N- and C-termini of each subunit appear to interact and are positioned at the base of the starch-binding groove extending away from each dimer in such a way that they may facilitate starch binding to the groove (**Figure 1**). However, this latter aspect of the model is more difficult to validate due to the apparent flexibility of both termini.

In order to test the validity of the tetrameric homology model of BAM2, we selected several conserved residues in the interfaces and changed them to residues that we expected would disrupt each interface. Once the proteins were purified we tested their activity and used SEC-MALS to determine their association state. Each mutant failed to form a tetramer and was instead either a dimer or a monomer, supporting the orientation of the BAM2 monomers in the model (**Figure 2C**). Interestingly, the two proteins with mutations in Interface A, necessary for forming the starch-binding groove, were nearly inactive, whereas the protein with a mutation in Interface B formed an active dimer (**Figure 2D**). Taken together, these results are consistent with the interpretation that BAM2 activity requires a dimer that contains the starch-binding groove, and that starch binding to the amino acids in the SBS does not stimulate formation of the dimer and the starch-binding groove. Why BAM2 forms a tetramer when a dimer is functional is a further question. These results also suggest that the tetramer interfaces in the model are a reasonably close approximation of the real BAM2 structure.

The requirement of K^+^ for BAM2 activity was surprising as no other BAMs are reported to be sensitive to salt levels and there is no evidence of catalytic metal ions in the known crystal structures. Because BAM2 is activated by a number of monovalent cations (Monroe et al., 2017), the interaction is most likely a Type II interaction in which the protein’s structure is altered by the presence of ions (Di Cera, 2006). Examination of the primary structure of BAM2 revealed an unusually high concentration of Glu and Asp residues in the 30-residue stretch between the chloroplast transit peptide and the catalytic domain, and an alignment with other BAM2 orthologs indicated that this “acidic region” was a feature of all BAM2s (**Figure 3**). Because these residues are expected to be negatively charged at physiological pH, their orientation relative to the TIM domain might be affected by the presence of cations. We were curious what effect removal of the acidic region would have on BAM2 activity and sensitivity to KCl, so we generated a mutant lacking the acidic region (NDel). Despite being located near Interface A and being part of the starch-binding groove (**Figure 1**), we were surprised to find that the NDel mutant was still a tetramer with considerable activity (**Figures 4C,D**). Importantly, NDel activity was nearly insensitive to KCl, and the reaction rate was higher than that of the WT protein at saturating KCl (**Figure 4D**). Because the effect of KCl on WT activity produces a sigmoidal curve (Monroe et al., 2017; **Figure 4D**) it seems likely that the interplay between the acidic region and K^+^, and the effect of starch binding to the groove in increasing amylase activity are somehow related. Further, the presence of this acidic region in BAM2 orthologs from all land plants and even in a charophyte alga, *Klebsormidium nitens* (**Figure 3**), suggests that it is involved in a highly conserved BAM2 function.

Close examination of the starch-binding groove revealed a centrally located Trp449 residue orientated such that the side chain ring might bond with a glucose residue of starch (**Figure 5C**). This type of aromatic-sugar stacking (π-interactions) is relatively common in sugar-binding proteins (Asensio et al., 2013) especially in SBS regions of starch hydrolases (Cuyvers et al., 2012; Cockburn et al., 2014). If this Trp residue is important for binding to starch, then we predicted that changing it to an Ala residue should result in a protein that is largely inactive, and that is what was observed (**Figure 5D**). Given the distance of this amino acid side chain from the active site, we do not believe that the activity defect is catalytic but is due to an inability to bind to starch efficiently to the starch-binding groove.

How and why starch binding to the starch-binding groove, facilitated by K^+^, activates BAM2 is most intriguing. Allosteric effectors can alter H-bond networks through proteins so we examined an alignment of BAM2 and BAM5 orthologs for conserved residues located between the active site and the starch-binding groove that differ between the two sets of proteins, to identify amino acids that may play a role in transmitting starch binding to the active site. One obvious difference was Ser464, which is adjacent to one of the two catalytic Glu residues in the active site (**Figure 6B**) and is proximal to the helix containing Trp449, which we had identified as important for starch binding to the starch-binding groove (**Figure 5**). The side chain of Ser464 faces away from the active site and toward the starch-binding groove (**Figure 6C**), and is conceivably involved in an allosteric interaction network. In all other catalytically active BAMs, this residue is a Gly. Changing Ser464 to Gly resulted in a protein that had a similar *V*_Max_ to the WT BAM2 (**Figures 6D,E**), but it displayed hyperbolic kinetics (Hill coefficient = 1) with a *K*_M_ for the substrate that was 7.5-fold lower than the WT protein (**Figures 6F,G**). The change in the Hill coefficient suggests that the S464G mutation disrupted the allosteric network between the starch-binding groove and the active site. Interestingly, the mutation resulted in an enzyme that was more efficient that the WT protein rather than less as might be expected. Previous studies on allosteric mutations within pyruvate kinase and phosphofructokinase indicate mutations to amino acids near allosteric sites can shift the equilibrium toward the T (inactive) or R (active) state (Heinisch et al., 1996; Valentini et al., 2000). Structures of these mutants can provide information on these conformations and structures of BAM2 in both the T and R states are necessary for further understanding of our findings and the role of Ser464. Overall, these data suggest that the allosteric network for BAM2 favors the T or inactive state unless the proper conditions such as ionic strength and starch binding are present.

How the unusual features of BAM2 are related to its physiological function is as-of-yet unknown, but we can speculate on what they might mean. Because of the cooperative kinetics, BAM2 activity is negligible at low concentrations of soluble starch. We attempted to assay BAM2 with amylose and amylopectin as the substrate, but their relative insolubility at high concentrations made these assays challenging. Soluble starch is rather different from the surface of a starch granule so it is difficult to predict the properties of BAM2 *in vivo*, but it is possible that BAM2 activity is also negligible at low levels of starch, which are known to occur at the end of the night. If BAM2 is present at this time of the photoperiod, it may be advantageous to prevent it from acting on small, growing molecules of starch. Moreover, this activity might be regulated by a yet unidentified metabolic, signaling, or post-translational modification pathway, which controls the T-R equilibrium of the protein. A possible function for the SBS is involvement in the separation of double helical strands of amylopectin, not unlike the role of an SBS on the surface of β-agarase that helps to separate double-helical chains of agarose (Allouch et al., 2004). If BAM2 acts in this way it might not require the enzymes that are known to facilitate the separation of double-helical chains of amylopectin, namely glucan water dikinase and phosphoglucan water dikinase (Hejazi et al., 2008). One argument against this possibility is that BAM2 is strongly coexpressed with glucan water dikinase (Monroe et al., 2017).

β-Amylases (BAMs) play an important role in starch degradation in plants. Flowering plants contain several active BAMs although their specific roles are not well understood. T-DNA mutants of Arabidopsis have been used to show that BAM3 plays a prominent role in leaf mesophyll cells at night, and that BAM1 plays a prominent role in guard cells across the night-day transition (Kaplan and Guy, 2005; Fulton et al., 2008; Valerio et al., 2011; Monroe et al., 2014; Horrer et al., 2016). In contrast, T-DNA mutants lacking BAM2 have no obvious phenotype in young leaves (Fulton et al., 2008), although older leaves of this mutant appear to accumulate starch (Monroe et al., 2014). Of interest is the observation that the first land plants contained at least two types of BAMs, at least one similar to BAM2 and at least one similar BAM1/3 (Monroe et al., 2017), suggesting that they may be complementary in function. The fact that BAM2 and BAM3 are quite different in their structural and catalytic properties may be a reflection of the dual nature of starch itself, being composed of amylose and amylopectin. Perhaps these enzymes act preferentially on one of the two polymers, and work synergistically to degrade starch efficiently. However, whereas BAM2 appears to be ancient and found in all basal land plant lineages, it is apparently lacking from several flowering plant families including the Solanaceae, Fabaceae, and Cucurbitaceae. It will be interesting to compare members of these groups to plants that possess BAM2 with respect to starch structure and metabolism. One thing is certain – we still have much to learn about the metabolism of this complex molecule.

## Author Contributions

JM, LP, JB, and AS designed and conducted the experiments. CB carried out the modeling. JM, CB, and AS wrote the manuscript.

## Conflict of Interest Statement

The authors declare that the research was conducted in the absence of any commercial or financial relationships that could be construed as a potential conflict of interest.
